# Project ImPACT Reduces Social Hyporesponsiveness and Translates to More Optimal Expressive Language Outcomes in Some Infants at Increased Likelihood of Autism

**DOI:** 10.1007/s10803-025-06928-3

**Published:** 2025-06-23

**Authors:** Jennifer E. Markfeld, Jacob I. Feldman, Catherine T. Bush, Paul J. Yoder, Tiffany G. Woynaroski

**Affiliations:** 1 Department of Hearing and Speech Sciences, Vanderbilt University, Nashville, TN USA; 2 Frist Center for Autism and Innovation, Vanderbilt University, Nashville, TN USA; 3 Department of Hearing and Speech Sciences, Vanderbilt University Medical Center, Nashville, TN USA; 4 Department of Special Education, Vanderbilt University, Nashville, TN USA; 5 Vanderbilt Kennedy Center, Vanderbilt University Medical Center, Nashville, TN USA; 6 Vanderbilt Brain Institute, Vanderbilt University, Nashville, TN USA; 7 Department of Communication Sciences and Disorders, John A. Burns School of Medicine, University of Hawaii at Manoa, Honolulu, HI USA

**Keywords:** Autism, Preemptive intervention, Prevention, Language, Sensory, Baby sibs

## Abstract

Low responsiveness to sensory stimuli, particularly stimuli that are social in nature (i.e., social hyporesponsiveness), predicts expressive language in autistic children and in infant siblings of autistic children (Sibs-autism), who are at high likelihood for a future diagnosis of autism and developmental language disorder. However, our understanding of whether social hyporesponsiveness can be addressed via early intervention to improve expressive language outcomes of Sibs-autism is limited. This randomized controlled trial investigated whether Project ImPACT, a caregiver-implemented Naturalistic Developmental Behavioral Intervention (NDBI), has an indirect effect on expressive language outcomes by reducing social hyporesponsiveness.  Sibs-autism were randomized into a Project ImPACT group (*n* = 23) for 12 weeks of intervention, or into a non-Project ImPACT control group (*n* = 23). Social hyporesponsiveness was measured immediately following intervention, and expressive language was measured three months after the end of intervention. Project ImPACT indirectly influenced distal expressive language outcomes through social hyporesponsiveness, but only for infants whose caregivers had high levels of education at study entry. Clinical implications of the results are discussed.

## Project ImPACT Reduces Social Hyporesponsiveness and Translates to More Optimal Expressive Language Outcomes in Some Infants at Increased Likelihood of Autism

Autism is a neurodevelopmental condition that is characterized by the presence of restricted and repetitive patterns of behaviors, interests, and activities as well as differences in social communication, and that commonly co-occurs with developmental language disorder (American Psychiatric Association [APA], 2013). Infant siblings of autistic children (Sibs-autism) are at high likelihood (i.e., one in five) for receiving a future diagnosis of autism, as well as for language delay and disorder, even when they are not on the autism spectrum (e.g., Charman et al., 2017; Landa et al., 2012; Marrus et al., 2018; Messinger et al., 2015; Ozonoff et al., 2011). Research and theory suggest that early differences in responding to sensory stimuli, specifically elevated social hyporesponsiveness, which is marked by decreased responding to sensory stimuli that are social in nature, may cascade onto expressive language acquisition in autistic children (e.g., Baranek et al., 2013; Bradshaw et al., 2022; Cascio et al., 2016; Watson et al., 2011). Emerging research suggests that social hyporesponsiveness may be increased in Sibs-autism and could influence the development of higher-order skills such as language in this high likelihood population as well (Baranek et al., 2018; Damiano-Goodwin et al., 2018; Feldman et al., 2021, 2022; Gunderson et al., 2021).

## The Rationale for Focusing on Infant Siblings of Autistic Children

A limitation of much of the extant literature, however, is that it has not historically focused on the sequelae of *early* differences in social hyporesponsiveness associated with autism, predominantly because autism cannot always be reliably diagnosed early in life (i.e., in infancy; Ozonoff et al., 2015, 2018; Woolfenden et al., 2012) and because research on Sibs-autism in this area has been limited to date. Therefore, the cascading effects framework conceptualizing the potential effects of social hyporesponsiveness on later expressive language has not been tested in infancy and early toddlerhood. The present work aims to assess these hypothesized cascading effects in this population.

## Preliminary Evidence that Sensory Responsiveness May be Malleable in Infants at Elevated Likelihood for Autism

Additionally, there is preliminary evidence suggesting that early, caregiver-mediated interventions may improve sensory hyporesponsiveness and yield gains in several distal developmental outcomes, including expressive language, in at least some infants at increased likelihood for autism (i.e., infants identified as being at high likelihood via broad-based community screening; Baranek et al., 2015). These promising findings suggest that focusing intervention efforts on improving sensory hyporesponsiveness may yield downstream effects on later expressive language in Sibs-autism. However, more work is needed to better understand whether early interventions could impact sensory hyporesponsiveness—particularly social hyporesponsiveness—and to determine whether decreasing social hyporesponsiveness results in improved expressive language outcomes, specifically in infants at heightened familial likelihood for a future diagnosis of autism and developmental language disorder (that is, in Sibs-autism).

## Project ImPACT as a Candidate Intervention

One intervention that may reduce social hyporesponsiveness and translate to more optimal expressive language outcomes in Sibs-autism is Project ImPACT (*Im*proving *P*arents *A*s *C*ommunication *T*eachers). Project ImPACT is a Natural Developmental Behavioral Intervention (NDBI) that uses strategies, taught to caregivers by trained coaches, to promote children’s language and communication development within daily routines and activities (Ingersoll & Dvortcsak, 2010; Schreibman et al., 2015). There is evidence from multiple studies that Project ImPACT yields favorable effects on autistic children’s spontaneous language use and broader development (e.g., Barber et al., 2020; Ingersoll & Wainer, 2013; Ingersoll et al., 2017; Killmeyer & Kaczmarek, 2017); as such, Project ImPACT has been recognized as an evidence-based practice for autistic children (Steinbrenner et al., 2020).

## Empirical Support for Indirect Effects of Project ImPACT in Sibs-autism

Additionally, there is evidence suggesting that Project ImPACT has the potential to produce indirect effects on the expressive language outcomes of Sibs-autism. Specifically, Yoder et al., (2021a, 2021b) found that caregiver use of Project ImPACT strategies yielded improved expressive language outcomes in Sibs-autism, through caregiver strategy use and more proximal child skills such as intentional communication. However, studies exploring the efficacy of Project ImPACT for Sibs-autism have not yet examined potential effects of Project ImPACT on social hyporesponsiveness. The strategies that caregivers are taught to use in this intervention (e.g., face to face positioning, use of animation) could well influence social hyporesponsiveness, which may represent another mechanism by which Project ImPACT produces positive effects on expressive language of Sibs-autism.

## A Need to Consider Factors that May Moderate Project ImPACT Effects

Moreover, there is a need to further explore contextual factors that may moderate the effects of Project ImPACT on expressive language outcomes in Sibs-autism (Mailick et al., 2025). Prior reports have assessed the potential influence of some moderators of NDBI effects on outcomes of interest in infants at high likelihood for autism, but findings for moderated effects have been somewhat inconsistent (e.g., Hampton & Rodriguez, 2022). Specific to Project ImPACT in Sibs-autism, a previously observed moderator of Project ImPACT on later expressive language was child “risk” status (e.g., operationalized according to biological sex, scores on an autism risk screener, number of siblings with autism; Yoder et al., 2021a). One factor that is unexplored thus far, but that our team hypothesized a priori may influence the effects of Project ImPACT on social hyporesponsiveness is socioeconomic status (SES) as indexed by caregiver level of education, which could influence caregivers’ ability to learn to use Project ImPACT strategies that they can then apply across a range of everyday routines with their infant (Klinger et al., 2021; Schreibman et al., 2011; Short et al., 2019). Prior work suggests that caregivers with lower education levels may face several barriers to implementing NDBIs, such as decreased time with their child, increased work demands, or less knowledge of child language acquisition and the processes contributing to development (e.g., Leung et al., 2020; Rowe, 2008; Rowe et al., 2005). Thus, we hypothesized that caregiver education level could influence the effect of Project ImPACT on social hyporesponsiveness in the present work.

## Moderated Mediation Analysis as an Approach to Testing Hypothesized Mechanisms

**Fig. 1:**
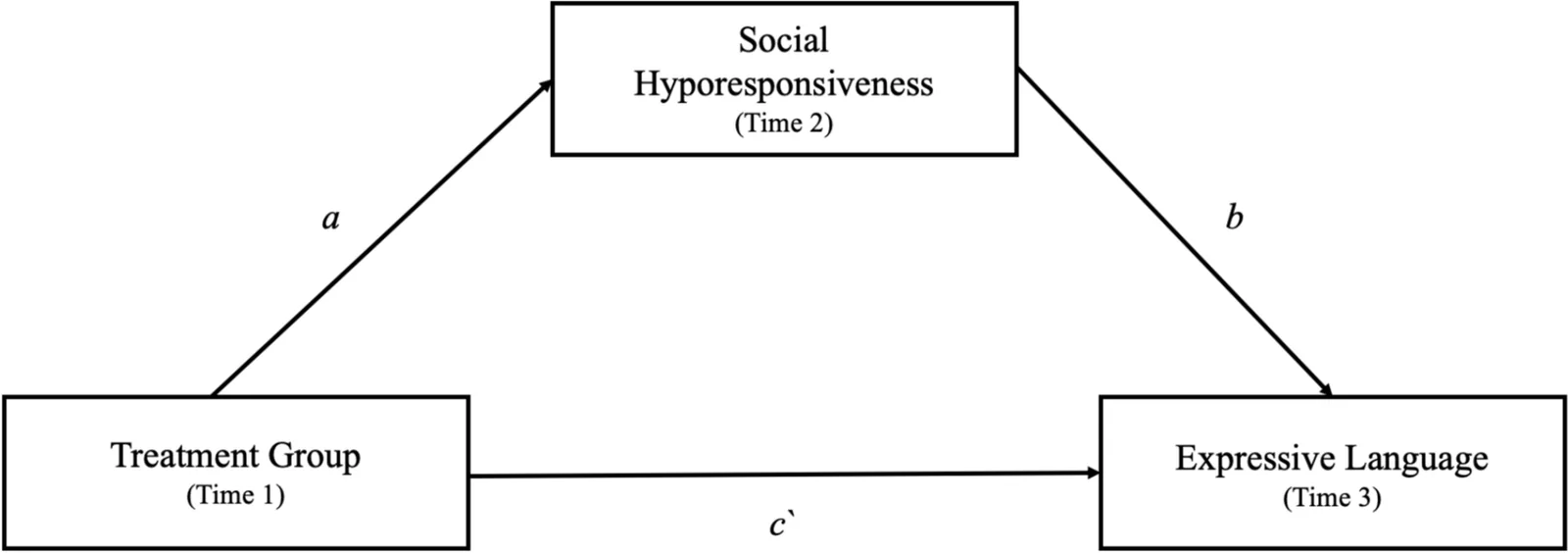
Figure depicts a conceptual mediation model to test the hypothesized indirect effect of Project ImPACT on later expressive language outcomes through social hyporesponsiveness. *a* = the relation between treatment group and social hyporesponsiveness; *b* = the relation between social hyporesponsiveness and later child language outcome, covarying treatment group; *c*′ = the direct effect of treatment group on later language outcomes, covarying social hyporesponsiveness

One approach to assess the mechanisms by which Project ImPACT may influence social hyporesponsiveness and translate to effects on expressive language outcomes of Sibs-autism is mediation analysis (Hayes, 2009). Through mediation analysis, we can assess the indirect effect of treatment group on expressive language outcomes through social hyporesponsiveness. This indirect effect comprises (a) the relation between treatment group and social hyporesponsiveness (i.e., the ‘*a*’ path), and (b) the relation between social hyporesponsiveness and expressive language outcomes, covarying for treatment group (i.e., the ‘*b*’ path). See Fig. 1 for a conceptual mediation model by which these paths can be tested. If the product of the coefficients for the *a* and *b* paths in the model is found to be significant, then we can conclude that there is evidence that social hyporesponsiveness mediates the relation between treatment group and expressive language outcomes. Further, in order to assess whether SES influences the hypothesized indirect effect of treatment group on expressive language through social hyporesponsiveness, moderation analyses can be conducted to evaluate whether SES influences the effect of Project ImPACT on social hyporesponsiveness.

## The Present Study

The present exploratory study, therefore, utilized a post hoc analytic approach to investigate whether Project ImPACT may reduce social hyporesponsiveness and translate to improved expressive language outcomes in Sibs-autism as compared to a non-Project ImPACT control group. Our specific research questions were as follows:

1. Does Project ImPACT produce greater reductions in social hyporesponsiveness in Sibs-autism relative to a non-Project ImPACT control? Is this effect moderated by SES (i.e., as indexed by caregiver education level)?
2. Does reduced social hyporesponsiveness translate to improved expressive language outcomes in Sibs-autism, controlling for treatment group?
3. Does social hyporesponsiveness mediate the association between treatment group (i.e., Project ImPACT versus non-Project ImPACT control) and later expressive language outcomes in Sibs-autism?

## Methods

All study procedures were approved by the Vanderbilt University Institutional Review Board. The research team comprises multiple caregivers of autistic children and clinicians who serve autistic individuals across the lifespan who participated in study conceptualization and execution (for complete details, see Community Involvement Statement).

## Participants

Participants were 46 Sibs-autism (20 male, 26 female), recruited for a larger randomized controlled trial of Project ImPACT (for more detailed information, see Yoder et al., 2021b; ClinicalTrials.gov ID: NCT03274622). All infants were between 12 and 18 months of age at study entry, had at least one autistic sibling, and lived in a primarily English-speaking home. Participants were recruited locally through print and digital advertisements (e.g., parent magazines, social media); flyers at parent advocacy and support groups, area pediatricians, schools and preschools, therapists and other providers for autistic children; and the Vanderbilt Kennedy Center. The social hyporesponsiveness measure was added to the battery of pre-registered instruments at one of the two sites after the larger trial began, when the last author generated the hypotheses relevant to this study. The present sample includes all of the participants from that single site who were enrolled beyond the timepoint when they could have completed this supplemental measure.

## Research Design

**Fig. 2:**
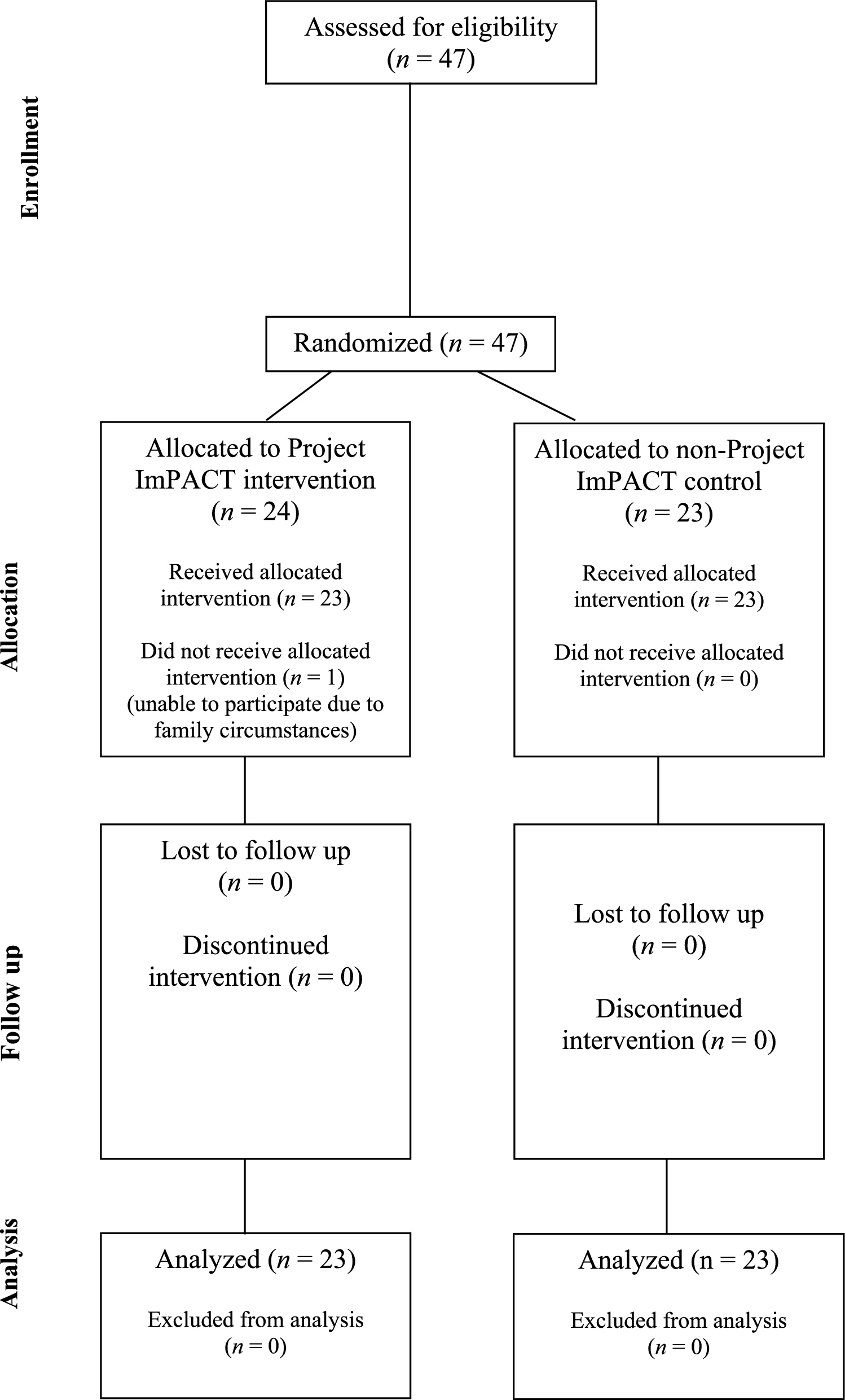
Figure depicts the CONSORT flow diagram for recruitment, randomization, enrollment, and retention of participants

Caregiver-child pairs were randomly assigned at study onset (Time 1) to either the Project ImPACT group (*n* = 23) or to the non-Project ImPACT control group (*n* = 23). See Fig. 2 for the CONSORT flow chart. Groups were well-matched on entry-level receptive language age, IQ, sex, and caregiver education level, *p* values for between-group differences at Time 1 ≥ 0.68. Groups also did not significantly differ on chronological age, mental age, or expressive language age at study entry, *p* values > 0.09. See Table 1 for a summary of descriptive statistics for Time 1 participant characteristics by intervention group.

**Table 1:** Participant characteristics by intervention group at study entry

	ImPACT *M* (*SD*)	Control *M* (*SD*)	*p*
Age (months)	13.85 (1.98)	14.64 (2.15)	0.20
Mental age (months)^a^	11.78 (2.15)	13.05 (2.62)	0.09
Expressive age (months)^a^	10.85 (3.31)	12.09 (3.67)	0.26
Receptive age (months)^a^	10.00 (2.87)	10.04 (2.69)	0.96
ELC	85.2 (16.5)	87.3 (16.2)	0.68
	*n*	*n*	
Biological Sex	9 Male 14 Female	11 Male 12 Female	0.55
Race	22 White 1 Multiple	19 White 2 Black or African American 2 Not Reported	0.21
Ethnicity	5 Hispanic or Latino 18 Not Hispanic or Latino	1 Hispanic or Latino 22 Not Hispanic or Latino	0.08
Primary Caregiver’s Highest Level of Education	3 High School Diploma or GED 4 College/Technical (1–2 yrs) 9 College/Technical (3–4 yrs) 3 Graduate/Professional School (1–2 yrs) 4 Graduate/Professional School (3 + yrs)	3 High School Diploma or GED 2 College/Technical (1–2 yrs) 13 College/Technical (3–4 yrs) 3 Graduate/Professional School (1–2 yrs) 2 Graduate/Professional School (3 + yrs)	0.72
Primary Caregiver	19 Mother 3 Father 1 Grandparent	20 Mother 3 Father 0 Other	0.58

*Note* ImPACT = Improving Parents as Communication Teachers; Control = Non-Project ImPACT control; ELC = Early Learning Composite, a commonly used proxy for IQ derived from the Mullen Scales of Early Learning (Mullen, 1995) and reported in standard scores (*M* = 100, *SD* = 15); GED = General Education Development. All between-group differences were non-significant at study entry

^a^Indexed via age equivalency scores on the Mullen Scales of Early Learning (Mullen, 1995). Mental age is derived as the average of age equivalency scores across receptive language, expressive language, fine motor, and visual reception scales

## Project ImPACT Intervention Group

Caregiver-child dyads assigned to the Project ImPACT group were offered 24 in-home teaching sessions over the course of 12 weeks with a speech-language pathologist (SLP) on the research team who had been trained and certified by the developer of Project ImPACT. During intervention sessions, caregivers were taught to use strategies intended to scaffold social communication, imitation, play, and language development. Some examples of Project ImPACT strategies include arranging the environment to encourage child engagement; ensuring face-to-face positioning; using animation; following the child’s lead; modeling and expanding language; using communicative temptations; and directly teaching skills such as language, motor imitation, and object play. Caregivers were instructed to use Project ImPACT strategies with their infant for at least 1 h per day, 5 days a week, for the duration of the study. Interventionist fidelity of coaching using the Project ImPACT protocol was calculated using self-administered checklists for at least two sessions per caregiver–child dyad and averaged 91% across sessions (*SD* = 4%). For further detail regarding Project ImPACT, see Ingersoll and Dvortcsak (2010).

## Non-Project ImPACT Control Group

The control group was not provided with Project ImPACT materials or training during the study. They were free to pursue intervention outside of the research study but did not choose to do so. Hours spent in non-project-related treatment did not significantly differ between groups, *t*(20) = − 1.00, *p* = 0.33 (Project ImPACT *M* = 0.1 h, *SD* = 0.44; non-Project ImPACT control *M* = 0 h, *SD* = 0). Thus, there was no evidence that community-based intervention or compensation could have influenced the outcomes of interest to this study.

## Measures

### Measure of Social Hyporesponsiveness

Immediately post-treatment (i.e., at Time 2; 3 months after study entry), social hyporesponsiveness was measured with a modified version of the Sensory Experiences Questionnaire (SEQ version 2.1; Baranek et al., 2006). The SEQ was selected as a psychometrically sound measure of the putative mediator in the early stages of the onset of the larger clinical trial (after the onset of, but prior to conducting analyses on, the larger trial) and was administered only at primary site (e.g., Baranek et al., 2006, 2013; Dakopolos & Jahromi, 2019; Little et al., 2011). The SEQ is a caregiver-report measure, intended for use with children ages 5 months to 6 years of age, that characterizes responses to everyday sensory stimuli in social and non-social contexts. Caregivers reported the frequency of occurrence of behaviors indexing specific patterns of sensory responsiveness on a 5-point Likert scale wherein Almost Never = 1, Once in a While = 2, Sometimes = 3, Frequently = 4, Almost Always = 5. Specifically, in this study caregivers answered selected questions from the SEQ tapping the pattern of sensory responsiveness of interest (e.g., “Does your child ignore you when you call his/her name?”, “Does your child ignore you (doesn’t notice) when you tap him/her on the shoulder for attention?”). For the purposes of this study, we derived the mean social hyporesponsiveness score for use in analyses (see Baranek et al., 2006, 2013 for more information on this measure and score). A higher mean score indicates a greater caregiver-reported frequency of behaviors associated with social hyporesponsiveness (i.e., higher scores indicate less noticing of or orienting to sensory stimuli that are social in nature such as the sound of their name being called and the feeling of being tapped on the shoulder). For further detail regarding the SEQ, see Baranek (1999b).

### Measures of Expressive Language

At the follow-up measurement period (i.e., Time 3; 6 months after study entry and 3 months after the SEQ was collected), multiple measures of expressive language that were pre-registered as primary outcome measures on the larger clinical trial were collected.

**Caregiver Report Measure**. Caregivers completed the MacArthur-Bates Communicative Development Inventories, Words and Sentences form (MCDI; Fenson et al., 2007), a caregiver-report measure of the words that their child understands and says. The total number of “words said” was derived for use in analyses.

**Observational Measures**. Child participants were directly assessed using the Communication and Symbolic Behavior Scales: Developmental Profile Behavior Sample (CSBS; Wetherby & Prizant, 2002), a semi-structured assessment of early language and communication development for children between 6–24 months of age; as well as the Brief Observation of Social Communication Change (BOSCC; Grzadzinski et al., 2016), a semi-structured assessment that is intended to measure core and related autism features over time. The location, interaction style, examiner, and materials used during the observational measures of expressive language notably differed from those used during intervention sessions; thus, these measures can be considered highly generalized. Examiners were also naïve to group assignment. For additional details on outcome measures, see Yoder et al. (2021b). The number of different words produced during the CSBS and BOSCC were derived for use in analyses.

### Measurement of SES

At study entry (Time 1), the primary caregiver of each Sibs-autism reported their highest level of education via a demographic form as a proxy for SES. Educational categories as listed in Table 1 were converted to a numerical scale for use in moderation analyses as follows: Elementary (0 years) = 1, Elementary (1–6 years) = 2, Elementary (7–9 years) = 3, Elementary (10–11 years) = 4, Elementary (12 years or General Educational Development test) = 5, College/Technical school (1–2 years) = 6, College/Technical school (3–4 years) = 7, Graduate/Professional school (1–2 years) = 8, Graduate/Professional school (3–4 + years) = 9.

## Analytic Plan

The analyses for the present study were not pre-registered; however, the following steps in our analytic plan were pre-planned. The plan to generate an aggregate from component variables derived from the measures of expressive language summarized above and the designation of the expressive language aggregate as a primary outcome was preregistered as part of the larger clinical trial (ClinicalTrials.gov ID: NCT03274622). The decision to impute missing data was made prior to analyses, but after the trial began.

Prior to analyses, data were assessed for normality, and variables that were not normally distributed (i.e., variables with skew >|1| or kurtosis >|3|) were transformed. The number of different words spoken during the CSBS and BOSCC and the total number of words said on the MCDI were transformed via square root transformation to correct for positive skew and kurtosis. The SEQ mean social hyporesponsiveness score was log10 transformed to correct for positive skew. Missing data (ranging from 1.1–6.5% across variables) were handled via imputation with the *missForest* package in R (R Core Team, 2022) to preserve power and reduce bias in an intent-to-treat approach to analysis (Enders, 2010; Stekhoven & Bühlmann, 2012). Missing data were imputed for all participants at the site where the SEQ was administered (i.e., participants from the other site were not included in these analyses).

In accord with the preregistered plan to increase the stability, and thus the potential construct validity, of expressive language outcomes (Rushton et al., 1983), an aggregate expressive language score was derived, following *z*-score transformation, from the following component variables: (a) the number of words said on the MCDI and (b) the number of different words spoken during the CSBS and the BOSCC (see Table 2). These variables were sufficiently intercorrelated to warrant aggregation (*r* values > 0.8 for all variables).

**Table 2:** Summary of key study constructs, measures, and variables according to research question

Construct	Measure(s)	Variable(s)	Role per research question	Measurement period
Social Hyporesponsiveness	SEQ	Mean social hyporesponsiveness score (log10 transformed)	DV (RQ 1), IV (RQ 2), Mediator (RQ 3)	Time 2
Later Expressive Language	MCDI CSBS BOSCC	Average of z scores for: (a) # words “child says” on MCDI (b) # different words spoken on CSBS (c) # different words spoken on BOSCC	DV (RQ 2, 3)	Time 3
Primary Caregiver’s Highest Level of Education	Demographic form	Highest level of education converted to numerical scale	Moderator (RQ 3)	Time 1

*Note.* RQ = research question; DV = dependent variable; IV = independent variable; SEQ = Sensory Experiences Questionnaire, version 2.1 (Baranek et al., 2006); MCDI = MacArthur-Bates Communicative Development Inventories, Words and Sentences (Fenson et al., 2007); CSBS = Communication and Symbolic Behavior Scales: Developmental Profile Behavior Sample (Wetherby & Prizant, 2002); BOSCC = Brief Observation of Social Communication Change (Grzadzinski et al., 2016); Time 1 = study entry; Time 2 = 3 months after study entry (i.e., at the end of intervention); Time 3 = 6 months after study entry (i.e., 3 months after caregiver training ended)

We ran a series of multiple regression analyses to answer our research questions. To answer our first research question, we ran a model assessing whether the effect of treatment group (i.e., Project ImPACT versus non-Project ImPACT) on social hyporesponsiveness was statistically significant (i.e., the* a* path relevant to the hypothesized indirect effect) and whether it was moderated by caregiver education level. To answer our second research question, we evaluated whether the association between social hyporesponsiveness and later expressive language outcomes was statistically significant, covarying for treatment group assignment (i.e., the *b* path relevant to the hypothesized indirect effect).

To answer our third research question, a mediation model was run using the PROCESS macro in R (Hayes, 2022; R Core Team, 2022) to evaluate whether social hyporesponsiveness mediated the effect of treatment group assignment (i.e., Project ImPACT versus non-Project ImPACT) on later expressive language outcomes. We included SES in the model when our previous analyses confirmed it was a significant moderator on the *a* path of interest. For all regression analyses, Cook’s D was utilized to monitor for undue influence, and Cohen’s *f*^2^ was utilized to quantify effect size (Selya et al., 2012).

## Results

### The Effect of Project ImPACT on Expressive Language Outcomes

The effect of treatment group on Time 3 expressive language (i.e., the unmediated effect of Project ImPACT on expressive language; Hayes, 2009) was not significant (β = –0.16, *p* = 0.30, Cohen’s *d* = 0.31).

## The Effect of Project ImPACT on Social Hyporesponsiveness

The effect of treatment group on social hyporesponsiveness (i.e., the *a* path relevant to the hypothesized indirect effect) was statistically significant (β = 1.87, *p* = 0.04, Cohen’s *f*^2^ = 0.10). However, this effect was significantly moderated by caregiver education level (β= - 2.04, *p* = 0.03, Cohen’s *f*^2^ = 0.12). To better understand this conditional effect, a Johnson-Neyman test was run to determine the cut-points along the continuous moderator (i.e., caregiver educational level) beyond which the treatment effect was significant. Results indicated that Project ImPACT was superior in reducing social hyporesponsiveness only in children whose caregivers had high levels of education (i.e., a score > 8.10 on our scale of caregiver level of education, which corresponds to approximately a masters-level education; 13.04% of the sample had an educational level above this cut-point).

## The Relation Between Social Hyporesponsiveness and Expressive Language Outcomes

The relation between social hyporesponsiveness and later child language, covarying for treatment group assignment (i.e., the *b* path relevant to the hypothesized indirect effect) was also statistically significant (β = –0.35, *p* = 0.02, Cohen’s *f*^2^ = 0.14). Children who presented with higher social hyporesponsiveness at Time 2 tended to have lower expressive language at T3, controlling for their treatment assignment. See Table 3 for a summary of results from the full regression models for the *a* and *b* paths.

**Table 3:** Summary of full regression models for paths comprising indirect effect

Model/Variable	*B* (SE)	*β*	*t*	*p*	*f* ^2^
*a* Path					
1. Constant	− 0.855 (0.518)	–	− 1.652	0.101	–
2. Treatment Group	1.419 (0.682)	1.867	2.080	0.044*	0.103
3. Caregiver Education	0.190 (0.074)	0.571	2.583	0.013*	0.159
4. Treatment Group × Caregiver Education	− 0.213 (0.096)	− 2.035	− 2.213	0.032*	0.117
*b* and *c’* paths					
1. Constant	0.550 (0.248)	–	2.213	0.032*	–
2. Sensory Hyporesponsiveness	− 0.860 (0.349)	− 0.349	− 2.464	0.0198*	0.141
3. Treatment Group	− 0.351 (0.266)	− 0.187	− 1.324	0.192	0.041

*Note* Coefficients, *p* values, and *f *^*2*^ values for regression analyses. *f*^2^ ≥.02 indicates a small effect size, *f*^2^ ≥.15 indicates a moderate effect size,* f*^2^ ≥.35 indicates a large effect size (Cohen, 1988). *a* path = the effect of treatment group on social hyporesponsiveness, as moderated by caregiver education level; *b* path = the effect of social hyporesponsiveness on expressive language, covarying for treatment group; *c′* path = the effect of treatment group on expressive language, covarying for the putative mediator (sensory hyporesponsiveness)

*p value for effect < 0.05

## Indirect Effect of Project ImPACT on Expressive Language Via Social Hyporesponsiveness

**Fig. 3:**
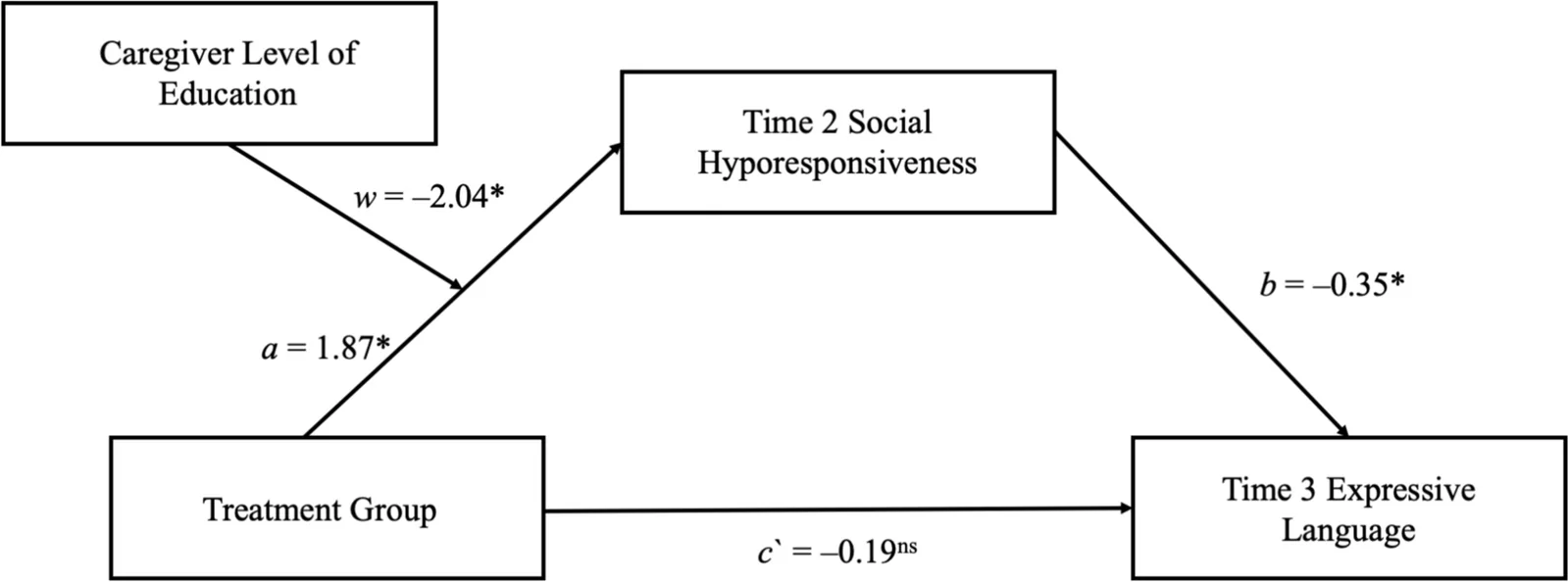
Figure depicts the moderated mediated relation between treatment group, social hyporesponsiveness, and expressive language according to caregiver highest level of education, our proxy for socioeconomic status. Treatment group = Project ImPACT or non-Project ImPACT control group; *a* = the effect of treatment group on social hyporesponsiveness as moderated by caregiver level of education (Baranek et al., 2006); *b* = the relation between social hyporesponsiveness and later child expressive language, covarying treatment group assignment; *c′* = the direct effect of treatment group on later expressive language, covarying social hyporesponsiveness. All values are standardized coefficients. *p < 0.05, *ns* = nonsignificant result.

We subsequently ran a moderated mediation model testing the effect of Project ImPACT on child expressive language outcomes through social hyporesponsiveness, retaining caregiver education as a moderator of the effect of Project ImPACT on social hyporesponsiveness (i.e., of the *a* path). This conditional indirect effect was significant, indicating that Project ImPACT indirectly influenced better child expressive language outcomes via reduced mid-point social hyporesponsiveness for children whose caregivers had a high level of education (95% confidence interval for moderated mediation effect = [0.02, 0.44]; see Fig. 3).

## Discussion

This study evaluated the effects of Project ImPACT, a caregiver-implemented NDBI, relative to a non-Project ImPACT control condition on social hyporesponsiveness and later expressive language of Sibs-autism who were 12–18 months of age. We found no unmediated effect of Project ImPACT on downstream expressive language. However, Project ImPACT did reduce social hyporesponsiveness and translate to more optimal expressive language outcomes in infants who entered treatment with caregivers who had relatively high levels of education. This finding has implications for research, theory, and clinical practice.

## Implications for Research and Theory

Our findings lend additional empirical support to the cascading effects theory (Cascio et al., 2016), which posits that targeting foundational skills such as sensory responsiveness, particularly early in life, may “cascade onto” higher-order skills such as language. This study builds upon prior preemptive intervention work in Sibs-autism focused on caregivers as implementers of intervention (see Hampton & Rodriguez, 2022). Previously, Project ImPACT was observed to indirectly improve expressive language outcomes in Sibs-autism via other pivotal child skills such as intentional communication and motor imitation, as well as increased caregiver use of Project ImPACT strategies (Yoder et al., 2021a, 2021b). Here, we highlight another proximal child skill that explains Project ImPACT effects on expressive language for this population—social hyporesponsiveness. This finding suggests that some of the strategies implemented in Project ImPACT (e.g., face to face positioning, use of animation) improve orienting and responding to social sensory stimuli in the environment, and that this subsequently scaffolds language acquisition for Sibs-autism with well-educated caregivers. Future studies, involving larger samples and employing more complex statistical models are needed to ascertain whether the multiple child characteristics and caregiver factors that have been identified as mediators of Project ImPACT effects to date have added value in explaining how this intervention influences child language skills. Such studies could help to further elucidate the mechanisms by which Project ImPACT is effective for improving expressive language skills, even in the absence of an unmediated effect of the treatment on spoken language.

## Implications for Clinical Practice

Our finding that social hyporesponsiveness was altered as a result of Project ImPACT only in infants with highly educated caregivers requires additional consideration. It may be that caregivers with higher levels of education are best positioned to learn the strategies presented in Project ImPACT via a didactic format (e.g., review of published materials assigned as homework, discussion of strategies, practice with the provision of feedback). It may also be that caregivers who report higher levels of education, a commonly used proxy for SES, are most optimally resourced to subsequently implement Project ImPACT strategies within the context of everyday routines with their infant (e.g., have more free time and/or access to more supports that facilitate regular interaction with their infant). Given that many caregiver-mediated interventions show only modest total effects, considering potential moderators of treatment effects such as caregiver education level and broader indices of SES is important when working to extend and adapt NDBIs such as Project ImPACT to fit the goals and needs of high likelihood infants and their families (Hampton & Rodriguez, 2021; Klinger et al., 2021; Sandbank et al., 2020). These findings underscore the need to develop and test personalized approaches for families who may be facing various barriers to implementing intervention (e.g., Mailick et al., 2025).

## Strengths and Limitations

To our knowledge, this is the first study investigating the effects of a preemptive intervention on later language outcomes through social hyporesponsiveness in Sibs-autism and their caregivers who were trained in Project ImPACT strategies. There are many strengths of this work. First, this study was a rigorous RCT wherein Sibs-autism were randomly assigned to a treatment or non-treatment condition, and randomization was successful in producing groups that were similar at pre-treatment. We assessed proximal and distal outcomes using measures that were highly generalized, such as caregiver reports and communication sampling procedures that differed from the intervention sessions in the dimensions of context, examiner, materials, and activities. Additionally, assessors and coders of language outcomes derived via the CSBS and BOSCC were naïve to condition. Despite the MCDI not being naive to condition due to it being a caregiver report, aggregating this measure with the CSBS and BOSCC decreases the likelihood that our variable indexing expressive language was swayed by lack of masking. Our participant attrition was low, and intent-to-treat analyses were employed to evaluate the mechanisms by which Project ImPACT influenced the outcome of interest.

Although this work represents an important step in advancing our understanding of the efficacy of preemptive interventions for Sibs-autism, there are several limitations to consider. First, social hyporesponsiveness was measured using a caregiver-report (i.e., the SEQ). In this study, caregivers committed substantial time and effort to the study and were not naïve to children’s treatment assignment; thus, the caregiver report measures utilized in this study, including the SEQ and the MCDI, were subject to detection and performance bias. Future work should, therefore, strongly consider including alternative, observational measures of sensory responsiveness and expressive language that can be administered by masked examiners (e.g., the Sensory Processing Assessment; Baranek et al., 1999c) to reduce bias and increase our confidence in effects of interest.

Additionally, we utilized caregiver education level as a proxy for SES. Future studies of interventions in Sibs-autism could take a more holistic approach to considering other aspects of socioeconomic status (e.g., family income), as well as caregiver and family factors that may contribute to intervention implementation and moderate treatment effects (e.g., household size, other children in the home with developmental conditions). Beyond measuring moderators such as SES, there is a need for future studies to evaluate the potential barriers to learning and implementing Project ImPACT in families wherein caregivers have lower levels of education (e.g., Diemer et al., 2013; Duncan et al., 2017). Additional research elucidating the challenges to implementation in families could help researchers understand how interventionists can work to achieve comparable intervention effects across SES strata.

Further, our small sample size limited our ability to test more complex models (e.g., to consider previously identified mediators and/or moderators of Project ImPACT treatment effects in the same model). Our small sample size additionally influences the identification of the cut point of caregiver education level defining the region of significance. A larger sample size would provide more statistical power to more precisely identify the education level at which Project IMPACT influences social hyporesponsiveness and translates to more optimal expressive language in future studies.

Finally, the exploratory nature of the current study begs for future replication of the effects detected in this study. Although the clinical trial that we leveraged was pre-registered, the specific hypotheses that we tested in this supplemental study involving a subset of the trial sample at a single trial site were not. Future pre-registered studies investigating effects of Project ImPACT and other NDBIs on child outcomes should attempt to replicate and extend the present findings in a larger sample that is better powered to detect relations between treatment, sensory responsiveness, and language outcomes according to child and family characteristics.

## Conclusion

This study sought to investigate whether Project ImPACT was effective in reducing social hyporesponsiveness and improving later expressive language outcomes in infants with older autistic siblings. We found a significant indirect effect of treatment group assignment on distal and highly generalized expressive language outcomes through social hyporesponsiveness in Sibs-autism who were 12–18 months of age. Caregiver level of education moderated this indirect effect, such that Project ImPACT reduced social hyporesponsiveness and translated to more optimal language outcomes only for infants with highly educated caregivers. Future work testing the efficacy of preemptive interventions for Sibs-autism should aim to better understand the mechanisms underlying treatment effects as well as pertinent moderators of caregiver-implemented intervention effects on language and broader developmental outcomes for this population at high familial likelihood for autism and developmental language disorder.

## Community Involvement Statement

Tiffany Woynaroski, who participated in the study design, conceptualization, data analyses, interpretation, and writing of the manuscript, is the parent of an autistic adult. Jacob I. Feldman, who participated in the data analyses, interpretation, and writing of the manuscript is the parent of an autistic child. Although no autistic individuals were directly involved in the conduction of this study, the authors are committed to engaging with the autistic community via community events and partnerships as well as ongoing research studies in their laboratory.

## References

[CR1] Baranek, G. T. (1999a). Autism during infancy: A retrospective video analysis of sensory-motor and social behaviors at 9–12 months of age. *Journal of Autism and Developmental Disorders,**29*(3), 213–224. 10.1023/A:1023080005650 10425584

[CR2] Baranek, G. T., Boyd, B. A., Poe, M. D., David, F. J., Watson, L. R., & MacLean, W. E. (2007). Hyperresponsive sensory patterns in young children with autism, developmental delay, and typical development. *American Journal of Mental Retardation,**112*(4), 233–245. 10.1352/0895-8017(2007)112[233:HSPIYC]2.0.CO;2 17559291

[CR3] Baranek, G. T., David, F. J., Poe, M. D., Stone, W. L., & Watson, L. R. (2006). Sensory experiences questionnaire: Discriminating sensory features in young children with autism, developmental delays, and typical development. *Journal of Child Psychology and Psychiatry, and Allied Disciplines,**47*(6), 591–601. 10.1111/j.1469-7610.2005.01546.x 16712636

[CR4] Baranek, G. T., Watson, L. R., Boyd, B. A., Poe, M. D., David, F. J., & McGuire, L. (2013). Hyporesponsiveness to social and nonsocial sensory stimuli in children with autism, children with developmental delays, and typically developing children. *Development and Psychopathology,**25*(2), 307–320. 10.1017/S0954579412001071 PMC3641693 23627946

[CR5] Baranek, G. T., Woynaroski, T. G., Nowell, S., Turner-Brown, L., DuBay, M., Crais, E. R., & Watson, L. R. (2018). Cascading effects of attention disengagement and sensory seeking on social symptoms in a community sample of infants at-risk for a future diagnosis of autism spectrum disorder. *Developmental Cognitive Neuroscience,**29*, 30–40. 10.1016/j.dcn.2017.08.006 PMC6414208 28869201

[CR6] Barber, A. B., Swineford, L., Cook, C., & Belew, A. (2020). Effects of Project ImPACT parent-mediated intervention on the spoken language of young children with autism spectrum disorder. *Perspectives of the ASHA Special Interest Groups,**5*(3), 573–581. 10.1044/2020_PERSP-20-10005

[CR7] Ben-Sasson, A., Gal, E., Fluss, R., Katz-Zetler, N., & Cermak, S. A. (2019). Update of a meta-analysis of sensory symptoms in ASD: A new decade of research. *Journal of Autism and Developmental Disorders,**49*(12), 4974–4996. 10.1007/s10803019041800 31501953

[CR8] Billstedt, E., Gillberg, C., & Gillberg, C. (2005). Autism after adolescence: Population-based 13- to 22-year follow-up study of 120 individuals with autism diagnosed in childhood. *Journal of Autism and Developmental Disorders,**35*(3), 351–360. 10.1007/s10803-005-3302-5 16119476

[CR9] Bradshaw, J., Schwichtenberg, A. J., & Iverson, J. M. (2022). Capturing the complexity of autism: Applying a developmental cascades framework. *Child Development Perspectives,**16*(1), 18–26. 10.1111/cdep.12439 PMC9673985 36407945

[CR10] Brock, M. E., Freuler, A., Baranek, G. T., Watson, L. R., Poe, M. D., & Sabatino, A. (2012). Temperament and sensory features of children with autism. *Journal of Autism and Developmental Disorders,**42*(11), 2271–2284. 10.1007/s10803-012-1472-5 PMC3482115 22366913

[CR11] Bryson, S. E., Zwaigenbaum, L., Brian, J., Roberts, W., Szatmari, P., Rombough, V., & McDermott, C. (2007). A prospective case series of high-risk infants who developed autism. *Journal of Autism and Developmental Disorders,**37*(1), 12–24. 10.1007/s10803-006-0328-2 17211728

[CR12] Cascio, C. J., Woynaroski, T., Baranek, G. T., & Wallace, M. T. (2016). Toward an interdisciplinary approach to understanding sensory function in autism spectrum disorder. *Autism Research,**9*(9), 920–925. 10.1002/aur.1612 PMC5564205 27090878

[CR13] Choi, B., Nelson, C. A., Rowe, M. L., & Tager-Flusberg, H. (2020). Reciprocal influences between parent input and child language skills in dyads involving high- and low-risk infants for autism spectrum disorder. *Autism Research,**13*(7), 1168–1183. 10.1002/aur.2270 32003131

[CR14] Dakopolos, A. J., & Jahromi, L. B. (2019). Differences in sensory responses among children with autism spectrum disorder and typical development: Links to joint attention and social competence. *Infant and Child Development,**28*(1), Article e2117.

[CR15] Dawson, G., Osterling, J., Meltzoff, A. N., & Kuhl, P. (2000). Case study of the development of an infant with autism from birth to two years of age. *Journal of Applied Developmental Psychology,**21*(3), 299–313. 10.1016/S01933973(99)000428 PMC3650850 23667283

[CR16] Dawson, G., Toth, K., Abbott, R., Osterling, J., Munson, J., Estes, A., & Liaw, J. (2004). Early social attention impairments in autism: Social orienting, joint attention, and attention to distress. *Developmental Psychology,**40*(2), 271–283. 10.1037/0012-1649.40.2.271 14979766

[CR17] Diemer, M. A., Mistry, R. S., Wadsworth, M. E., López, I., & Reimers, F. (2013). Best practices in conceptualizing and measuring social class in psychological research. *Analyses of Social Issues and Public Policy,**13*(1), 77–113. 10.1111/asap.12001

[CR18] Eaves, L. C., & Ho, H. H. (2008). Young adult outcome of autism spectrum disorders. *Journal of Autism and Developmental Disorders,**38*(4), 739–747. 10.1007/s10803-007-0441-x 17764027

[CR19] Eisenberg, L. (1956). The autistic child in adolescence. *American Journal of Psychiatry,**112*(8), 607–612. 10.1176/ajp.112.8.607 13292547

[CR20] Enders, C. K. (2010). *Applied missing data analysis*. Guilford Press.

[CR21] Suzman, E., Augustine, A. E., Garla, V., Muhumuza, A., Cascio, C. J., Williams, K. L., Kirby, A. V., Keçeli-Kaysılı, B., & Woynaroski, T. G. (2021). Sensory responsiveness is linked with communication in infant siblings of children with and without autism. *Journal of Speech, Language, and Hearing Research,**64*(6), 1964–1976. 10.1044/2021_JSLHR-20-00196 PMC8740754 34003699

[CR22] Freuler, A., Baranek, G. T., Watson, L. R., Boyd, B. A., & Bulluck, J. C. (2012). Precursors and trajectories of sensory features: Qualitative analysis of infant home videos. *The American Journal of Occupational Therapy,**66*(5), e81–e84. 10.5014/ajot.2012.004465 PMC3428724 22917133

[CR23] Gillberg, C., & Steffenburg, S. (1987). Outcome and prognostic factors in infantile autism and similar conditions: A population-based study of 46 cases followed through puberty. *Journal of Autism and Developmental Disorders,**17*(2), 273–287. 10.1007/BF01495061 3610999

[CR24] Grzadzinski, R., Carr, T., Colombi, C., McGuire, K., Dufek, S., Pickles, A., & Lord, C. (2016). Measuring changes in social communication behaviors: Preliminary development of the Brief Observation of Social Communication Change (BOSCC). *Journal of Autism and Developmental Disorders,**46*(7), 2464–2479. 10.1007/s10803-016-2782-9 27062034

[CR25] Gunderson, J., Worthley, E., Grzadzinski, R., Burrows, C., Estes, A., Zwaigenbaum, L., Botteron, K., Dager, S., Hazlett, H., Schultz, R., Piven, J., Wolff, J., & Network, I. B. I. S. (2021). Social and non-social sensory responsivity in toddlers at high-risk for autism spectrum disorder. *Autism Research: Official Journal of the International Society for Autism Research,**14*(10), 2143–2155. 10.1002/aur.2556 PMC8487998 34145789

[CR26] Hampton, L. H., & Rodriguez, E. M. (2022). Preemptive interventions for infants and toddlers with a high likelihood for autism: A systematic review and meta-analysis. *Autism,**26*(6), 1364–1378. 10.1177/13623613211050433 34628968

[CR27] Hayes, A. F. (2009). Beyond Baron and Kenny: Statistical mediation analysis in the new millennium. *Communication Monographs,**76*(4), 408–420. 10.1080/03637750903310360

[CR28] Ingersoll, B. R. (2010). Teaching social communication: A comparison of naturalistic behavioral and development, social pragmatic approaches for children with autism spectrum disorders. *Journal of Positive Behavior Interventions,**12*(1), 33–43. 10.1177/1098300709334797

[CR29] Ingersoll, B., & Wainer, A. (2013). Initial efficacy of Project ImPACT: A parent-mediated social communication intervention for young children with ASD. *Journal of Autism and Developmental Disorders,**43*(12), 2943–2952. 10.1007/s10803-013-1840-9 23689760

[CR30] Ingersoll, B. R., Wainer, A. L., Berger, N. I., & Walton, K. M. (2017). Efficacy of low intensity, therapist-implemented Project ImPACT for increasing social communication skills in young children with ASD. *Developmental Neurorehabilitation,**20*(8), 502–510. 10.1080/17518423.2016.1278054 28152327

[CR31] Killmeyer, S., & Kaczmarek, L. (2017). Parent training and joint engagement in young children with autism spectrum disorder. *Autism & Developmental Language Impairments,**2*, 1–16. 10.1177/2396941517699214

[CR32] Kirby, A. V., Bilder, D. A., Wiggins, L. D., Hughes, M. M., Davis, J., Hall-Lande, J. A., Lee, L.-C., McMahon, W. M., & Bakian, A. V. (2022). Sensory features in autism: Findings from a large population-based surveillance system. *Autism Research,**15*(4), 751–760. 10.1002/aur.2670 PMC9067163 35040592

[CR33] Kobayashi, R., Murata, T., & Yoshinaga, K. (1992). A follow-up study of 201 children with autism in Kyushu and Yamaguchi areas, Japan. *Journal of Autism and Developmental Disorders,**22*, 395–411. 10.1007/BF01048242 1383189

[CR34] Landa, R. J., Gross, A. L., Stuart, E. A., & Bauman, M. (2012). Latent class analysis of early developmental trajectory in baby siblings of children with autism. *Journal of Child Psychology and Psychiatry, and Allied Disciplines,**53*(9), 986–996. 10.1111/j.1469-7610.2012.02558.x PMC3432306 22574686

[CR35] Liss, M., Saulnier, C., Fein, D., & Kinsbourne, M. (2006). Sensory and attention abnormalities in autistic spectrum disorders. *Autism,**10*(2), 155–172. 10.1177/1362361306062021 16613865

[CR36] Messinger, D., Young, G. S., Ozonoff, S., Dobkins, K., Carter, A., Zwaigenbaum, L., Landa, R. J., Charman, T., Stone, W. L., Constantino, J. N., Hutman, T., Carver, L. J., Bryson, S., Iverson, J. M., Strauss, M. S., Rogers, S. J., & Sigman, M. (2013). Beyond autism: A Baby Siblings Research Consortium study of high-risk children at three years of age. *Journal of the American Academy of Child & Adolescent Psychiatry,**52*(3), 300-308.e1. 10.1016/j.jaac.2012.12.011 PMC3625370 23452686

[CR37] Mullen, E. M. (1995). *Mullen scales of early learning*. American Guidance Service.

[CR38] Nowell, S. W., Watson, L. R., Crais, E. R., Baranek, G. T., Faldowski, R. A., & Turner-Brown, L. (2020). Joint attention and sensory-regulatory features at 13 and 22 months as predictors of preschool language and social-communication outcomes. *Journal of Speech, Language, and Hearing Research,**63*(9), 3100–3116. 10.1044/2020_JSLHR-20-00036 32810416

[CR39] Ozonoff, S., Gangi, D., Hanzel, E. P., Hill, A., Hill, M. M., Miller, M., Schwichtenberg, A. J., Steinfeld, M. B., Parikh, C., & Iosif, A.-M. (2018). Onset patterns in autism: Variation across informants, methods, and timing. *Autism Research,**11*(5), 788–797. 10.1002/aur.1943 PMC5992045 29524310

[CR40] Ozonoff, S., Young, G. S., Carter, A., Messinger, D., Yirmiya, N., Zwaigenbaum, L., Bryson, S., Carver, L. J., Constantino, J. N., Dobkins, K., Hutman, T., Iverson, J. M., Landa, R., Rogers, S. J., Sigman, M., & Stone, W. L. (2011). Recurrence risk for autism spectrum disorders: A Baby Siblings Research Consortium study. *Pediatrics,**128*(3), e488–e495. 10.1542/peds.2010-2825 PMC3164092 21844053

[CR41] Ozonoff, S., Young, G. S., Landa, R. J., Brian, J., Bryson, S., Charman, T., Chawarska, K., Macari, S. L., Messinger, D., Stone, W. L., Zwaigenbaum, L., & Iosif, A.-M. (2015). Diagnostic stability in young children at risk for autism spectrum disorder: A Baby Siblings Research Consortium study. *Journal of Child Psychology and Psychiatry, and Allied Disciplines,**56*(9), 988–998. 10.1111/jcpp.12421 PMC4532646 25921776

[CR42] Rowe, M. L. (2008). Child-directed speech: Relation to socioeconomic status, knowledge of child development and child vocabulary skill. *Journal of Child Language,**35*(1), 185–205. 10.1017/S0305000907008343 18300434

[CR43] Rowe, M. L., Pan, B. A., & Ayoub, C. (2005). Predictors of variation in maternal talk to children: A longitudinal study of low-income families. *Parenting,**5*(3), 259–283. 10.1207/s15327922par0503_3

[CR44] Rushton, J. P., Brainerd, C. J., & Pressley, M. (1983). Behavioral development and construct validity: The principle of aggregation. *Psychological Bulletin,**94*, 18–38. 10.1037/0033-2909.94.1.18

[CR45] Sabatos-DeVito, M., Schipul, S. E., Bulluck, J. C., Belger, A., & Baranek, G. T. (2016). Eye tracking reveals impaired attentional disengagement associated with sensory response patterns in children with autism. *Journal of Autism and Developmental Disorders,**46*(4), 1319–1333. 10.1007/s10803-015-2681-5 PMC5359772 26816345

[CR46] Watson, L. R., Patten, E., Baranek, G. T., Poe, M., Boyd, B. A., Freuler, A., & Lorenzi, J. (2011). Differential associations between sensory response patterns and language, social, and communication measures in children with autism or other developmental disabilities. *Journal of Speech, Language, and Hearing Research,**54*(6), 1562–1576. 10.1044/1092-4388(2011/10-0029) PMC3325756 21862675

[CR47] Wetherby, A. M., & Prizant, B. M. (2002). *Communication and symbolic behavior scales: Developmental profile*. Brookes Publishing Co.

[CR48] Woolfenden, S., Sarkozy, V., Ridley, G., & Williams, K. (2012). A systematic review of the diagnostic stability of autism spectrum disorder. *Research in Autism Spectrum Disorders,**6*(1), 345–354. 10.1016/j.rasd.2011.06.008

[CR49] Yoder, P. J., Stone, W. L., & Edmunds, S. R. (2021a). For which younger siblings of children with ASD does parent-mediated intervention work? *Autism,**25*(1), 58–69. 10.1177/1362361320943373 PMC7854964 32811171

[CR50] Yoder, P. J., Stone, W. L., & Edmunds, S. R. (2021b). Parent utilization of ImPACT intervention strategies is a mediator of proximal then distal social communication outcomes in younger siblings of children with ASD. *Autism,**25*(1), 44–57. 10.1177/1362361320946883 PMC7854804 32811160

[CR51] American Psychiatric Association. (2013). *Diagnostic and statistical manual of mental disorders* (5^th^ ed.). 10.1176/appi.books.9780890425596

[CR52] Baranek, G. T. (1999b).&nbsp;*Sensory Experiences Questionnaire (SEQ)*&nbsp;(Version 2.1) [Unpublished manuscript]. University of North Carolina at Chapel Hill.

[CR53] Baranek, G. T. (1999c). *Sensory processing assessment for young children (SPA)* [Unpublished manuscript]. University of North Carolina at Chapel Hill.

[CR54] Baranek, G. T., Watson, L. R., Turner-Brown, L., Field, S. H., Crais, E. R., Wakeford, L., Little, L. M., & Reznick, J. S. (2015). Preliminary efficacy of adapted responsive teaching for infants at risk of autism spectrum disorder in a community sample. *Autism Research and Treatment*, *2015*, Article 386951. 10.1155/2015/386951 PMC4306223 25648749

[CR55] Bradshaw, J., Steiner, A. M., Gengoux, G., & Koegel, L. K. (2014). Feasibility and effectiveness of very early intervention for infants at-risk for autism spectrum disorder: A systematic review. *Journal of Autism and Developmental Disorders*, *45*, 778–794. 10.1007/s10803-014-2235-2 25218848

[CR56] Charman, T., Loth, E., Tillmann, J., Crawley, D., Wooldridge, C., Goyard, D., Ahmad, J., Auyeung, B., Ambrosino, S., Banaschewski, T., Baron-Cohen, S., Baumeister, S., Beckmann, C., Bölte, S., Bourgeron, T., Bours, C., Brammer, M., Brandeis, D., Brogna, C., … Buitelaar, J. K. (2017). The EU-AIMS Longitudinal European Autism Project (LEAP): Clinical characterisation. *Molecular Autism*, *8*(1), Article 27. 10.1186/s13229-017-0145-9 PMC5481972 28649313

[CR57] Duncan, G. J., Magnuson, K., & Votruba-Drzal, E. (2017). Moving beyond correlations in assessing the consequences of poverty. *Annual Review of Psychology, 68,* 413–434.&nbsp; 10.1146/annurev-psych-010416-044224 PMC6108837 27648987

[CR58] Feldman, J. I., Cassidy, M., Liu, Y., Kirby, A. V., Wallace, M. T., & Woynaroski, T. G. (2020). Relations between sensory responsiveness and features of autism in children. *Brain Sciences*, *10*(11), Article 775. 10.3390/brainsci10110775 PMC7690864 33114357

[CR59] Feldman, J. I., Raj, S., Bowman, S. M., Santapuram, P., Golden, A. J., Daly, C., Dunham, K.,

[CR60] Fenson, L., Marchman, V. A., Thal, D. J., Dale, P. S., & Reznick, J. S. (2007). *MacArthur-Bates communicative development inventories: User’s guide and technical manual*. Brookes Publishing.

[CR61] Hayes, A. F. (2022). *Introduction to mediation, moderation, and conditional process analysis: A regression-based approach* (3rd ed.). Guilford Press.

[CR62] Ingersoll, B., & Dvortcsak, A. (2010). *Teaching social communication to children with autism: A practitioner’s guide to parent training and a manual for parents*. Guilford Press.

[CR63] Klinger, L. G., Cook, M. L., & Dudley, K. M. (2021). Predictors and moderators of treatment efficacy in children and adolescents with autism spectrum disorder.&nbsp;*Journal of Clinical Child & Adolescent Psychology*,&nbsp;*50*(4), 517–524. 10.1080/15374416.2020.1833735 PMC8986328 33210939

[CR64] Leung, C. Y. Y., Hernandez, M. W., & Suskind, D. L. (2020). Enriching home language environment among families from low-SES backgrounds: A randomized controlled trial of a home visiting curriculum. *Early Childhood Research Quarterly*, *50*, 24–35. 10.1016/j.ecresq.2018.12.005

[CR65] Mailick, M., Bennett, T., DaWalt, L. S., Durkin, M. S., Forbes, G., Howlin, P., Lord, C., Zaidman-Zait, A., Zwaigenbaum, L., Bal, V., Bishop, S., Chiang, C., DiMartino, A., Freitag, C. M., Georgiades, S., Hollocks, M., Lai, M., Maenner, M. J., Powell, P. S., Lounds Taylor, J., & Halladay, A. (2025). Expanding research on contextual factors in autism research: What took us so long?.&nbsp;*Autism Research*. Advance online publication. 10.1002/aur.3312 PMC12015804 39902495

[CR66] Marrus, N., Hall, L. P., Paterson, S. J., Elison, J. T., Wolff, J. J., Swanson, M. R., Parish-Morris, J., Eggebrecht, A. T., Pruett, J. R., Hazlett, H. C., Zwaigenbaum, L., Dager, S., Estes, A. M., Schultz, R. T., Botteron, K. N., Piven, J., Constantino, J. N., & IBIS Network. (2018). Language delay aggregates in toddler siblings of children with autism spectrum disorder. *Journal of Neurodevelopmental Disorders*, *10*(1), Article 29. 10.1186/s11689-018-9247-8 PMC6198516 30348077

[CR67] Messinger, D. S., Young, G. S., Webb, S. J., Ozonoff, S., Bryson, S. E., Carter, A., Carver, L., Charman, T., Chawarska, K., Curtin, S., Dobkins, K., Hertz-Picciotto, I., Hutman, T., Iverson, J. M., Landa, R., Nelson, C. A., Stone, W. L., Tager-Flusberg, H., & Zwaigenbaum, L. (2015). Early sex differences are not autism-specific: A Baby Siblings Research Consortium (BSRC) study. *Molecular Autism*, *6*(1), Article 32. 10.1186/s13229-015-0027-y PMC4455973 26045943

[CR68] Pecukonis, M., Young, G. S., Brian, J., Charman, T., Chawarska, K., Elsabbagh, M., & Tager‐ Flusberg, H. (2022). Early predictors of language skills at 3 years of age vary based on diagnostic outcome: A Baby Siblings Research Consortium study. *Autism Research, 15*(7), 1324–1335. 10.1002/aur.2760 PMC9253079 35652157

[CR69] R Core Team. (2022). R: A language and environment for statistical computing (Version 4.2.2). R Foundation for Statistical Computing: Vienna, Austria. https://www.R-project.org/

[CR70] Roberts, M. Y., Curtis, P. R., Sone, B. J., & Hampton, L. H. (2019). Association of parent training with child language development: A systematic review and meta-analysis. *JAMA Pediatrics*, *173*(7), 671–680. 10.1001/jamapediatrics.2019.1197 PMC6537769 31107508

[CR71] Schreibman, L., Dufek, S., spsampsps Cunningham, A. B. (2011). Identifying moderators of treatment outcome for children with autism. Inspsampspsnbsp;*International handbook of autism and pervasive developmental disorders*spsampspsnbsp;(pp. 295–305). Springer New York.

[CR72] Selya, A. S., Rose, J. S., Dierker, L. C., Hedeker, D., & Mermelstein, R. J. (2012). A practical guide to calculating Cohen’s f^2^, a measure of local effect size, from PROC MIXED. *Frontiers in Psychology*, *3*, Article 111. 10.3389/fpsyg.2012.00111 PMC3328081 22529829

[CR73] Short, K., Eadie, P., & Kemp, L. (2019). Paths to language development in at risk children: A qualitative comparative analysis (QCA). *BMC Pediatrics*, *19*(1), Article 94. 10.1186/s12887-019-1449-z PMC6449893 30953552

[CR74] Steinbrenner, J. R., Hume, K., Odom, S. L., Morin, K. L., Nowell, S. W., Tomaszewski, B., Szendrey, S., McIntyre, N. S., Yücesoy-Özkan, S., & Savage, M. N. (2020). *Evidence-based practices for children, youth, and young adults with autism*. The University of North Carolina at Chapel Hill, Frank Porter Graham Child Development Institute, National Clearinghouse on Autism Evidence and Practice Review Team. https://ncaep.fpg.unc.edu/sites/ncaep.fpg.unc.edu/files/imce/documents/EBP%20Report%202020.pdf

[CR75] Yoder, P., Watson, L. R., & Lambert, W. (2015). Value-added predictors of expressive and receptive language growth in initially nonverbal preschoolers with autism spectrum disorders.&nbsp;*Journal of Autism and Developmental Disorders*,&nbsp;*45*(5), 1254–1270. 10.1007/s10803-014-2286-4 PMC4495651 25344152

